# Exploring the Next Frontier for Tobacco Control: Nondaily Smoking among New York City Adults

**DOI:** 10.1155/2012/145861

**Published:** 2012-05-20

**Authors:** Rachel Sacks, Micaela H. Coady, Ijeoma G. Mbamalu, Michael Johns, Susan M. Kansagra

**Affiliations:** Bureau of Chronic Disease Prevention and Tobacco Control, New York City Department of Health and Mental Hygiene, CN-18, Queens, NY 11101, USA

## Abstract

*Objective*. Among current smokers, the proportion of Nondaily smokers is increasing. A better understanding of the characteristics and smoking behaviors of Nondaily smokers is needed. *Methods*. We analyzed data from the New York City (NYC) Community Health Survey to explore Nondaily smoking among NYC adults. Univariate analyses assessed changes in Nondaily smoking over time (2002–2010) and identified unique characteristics of Nondaily smokers; multivariable logistic regression analysis identified correlates of Nondaily smoking in 2010. *Results*. The proportion of smokers who engage in Nondaily smoking significantly increased between 2002 and 2010, from 31% to 36% (*P* = 0.05). A larger proportion of Nondaily smokers in 2010 were low income and made tax-avoidant cigarette purchases compared to 2002. Smoking behaviors significantly associated with Nondaily smoking in 2010 included smoking more than one hour after waking (AOR = 8.8, 95% CI (5.38–14.27)); buying “loosies” (AOR = 3.5, 95% CI (1.72–7.08)); attempting to quit (AOR = 2.3, 95% CI (1.36–3.96)). *Conclusion*. Nondaily smokers have changed over time and have characteristics distinct from daily smokers. Tobacco control efforts should be targeted towards “ready to quit” Nondaily smokers.

## 1. Introduction

Nondaily smoking, also referred to as intermittent or occasional smoking, represents a new challenge for tobacco research and control, both nationally and in New York City (NYC). Expanding smoke-free environments and higher cigarette taxes have been associated with declines in daily smoking nationwide and on a state-by-state basis [1–4]. However, alongside this downward trend in daily smoking, Nondaily smoking has risen. Between 2002 and 2010, the proportion of Nondaily smokers within the US adult smoker population rose [5, 6].

A better understanding of the Nondaily smoking population is needed in order to inform the development of educational efforts and cessation interventions that address this shift in the smoking population [7–10]. Previous studies have characterized Nondaily smoking as either an indicator of a tobacco initiation period common among college students and young adults [11, 12], a transition stage among daily smokers that precedes cessation [13–15], or a stable, long-term smoking behavior [1, 13]. Due to their high rates of quit attempts [8, 16], Nondaily smokers are also seen as a “ready to quit” subgroup of smokers that could benefit from cessation advice [7, 17]. Yet tobacco control programs and healthcare providers may be overlooking these smokers, either because Nondaily smokers do not self-identify as “smokers” [1, 2, 13], do not perceive themselves at risk for the negative health consequences of smoking [1, 7, 17], or may be ineligible for cessation programs that provide pharmacotherapy [18].

Communicating the dangers of smoking to Nondaily smokers is further complicated by the existence of subgroups within this population. Previous studies have described Nondaily smokers as younger, predominantly female, better educated, and wealthier than daily smokers [14, 19]. Research has also found greater representation of racial/ethnic minorities among Nondaily smokers as compared to daily smokers [7, 17, 20], and particularly high levels of Nondaily smoking among Hispanics [21]. However, these broad demographic categories may not provide sufficient information to inform targeted cessation interventions for Nondaily smokers, especially considering the diversity of smoking behaviors seen in this population [1, 7]. The so-called social smokers who smoke primarily in social situations have been the subject of exploratory research that has identified associations between smoking and binge drinking, especially among college students [11, 22, 23]. By contrast, former daily smokers who have reduced their smoking in response to tax increases or smoke-free air laws represent a different subgroup of the Nondaily smoking population that may be older and more sensitive to tobacco control policies than social smokers [24–26].

NYC provides an ideal environment in which to examine a diverse population of smokers to both assess Nondaily smoking over time and more closely examine the demographic and smoking characteristics of Nondaily smokers. In 2002, NYC launched a comprehensive tobacco control plan that included (1) taxation, including four cigarette tax increases since 2002; (2) legislation, which rendered workplaces smoke-free, including restaurants and bars; (3) expansion of treatment options for smokers via provision of nicotine replacement therapy for daily smokers; (4) intensive antitobacco education via various media channels. After implementing this plan, the prevalence of adult smoking in NYC dropped significantly from 22% in 2002 to 16% in 2009 [27].

Using a population-based survey of NYC adults, our objectives were threefold: (1) to assess whether the proportion and characteristics of Nondaily smokers have changed between 2002 and 2010; using the 2010 data only, (2) to compare the demographic characteristics, and smoking behaviors of different types of smokers (Nondaily, light daily and heavy daily); (3) to explore characteristics associated with Nondaily smoking.

## 2. Materials and Methods

### 2.1. Data Collection and Sample

Nondaily smoking data were collected using the NYC Community Health Survey (CHS), a population-based, random-digit-dialed telephone survey of approximately 10,000 NYC adults, aged 18 or older. The NYC DOHMH has conducted the CHS annually since 2002. The survey is available in multiple languages, including Spanish, Russian, and Chinese. All interviews were conducted by trained interviewers.

In 2002, eligible households were contacted using landlines only. A total of 9,674 interviews were conducted, representing a 36% response rate and a 69% cooperation rate among contacted households [28]. In 2010, landlines and cell phone numbers were used to contact potential respondents, resulting in 8,665 interviews. Response and cooperation rates of 34% and 88% for landline users and 46% and 96% for cell phone users were achieved among those contacted.

### 2.2. Instrument

The NYC CHS instrument was adapted from the Centers for Disease Control's Behavioral Risk Factor Surveillance System (BRFSS) [29]. The tobacco module includes questions related to current smoking, secondhand smoke exposure, responses to increases in taxation of tobacco products, and smoking cessation.

Current smoking was defined as presently smoking on all or some days and having smoked at least 100 cigarettes in a lifetime; “Nondaily” smoking was defined as smoking on some days. Daily smokers were classified as “heavy” or “light” depending on the number of cigarettes smoked per day (CPD). Heavy smoking was defined as 11 or more CPD; light smoking as 10 or less CPD. Respondents who reported smoking 11 or more CPD on some days were classified as heavy smokers (19 cases in 2002 and 4 cases in 2010).

Missing CPD data was imputed (40 cases in 2002, 69 cases in 2010) using mean replacement. In 2002, the mean number of cigarettes per day was calculated using only everyday smokers. In 2010, respondents were first asked how many cigarettes they smoked on the days they smoked and then asked how many days per month they smoked. When the number of cigarettes smoked was available but number of days smoked was missing, the days smoked were replaced with the mean of days smoked for all Nondaily smokers. If both the number of cigarettes smoked and days smoked were missing, then values were imputed based on the mean for Nondaily smokers.

A quit attempt was defined as intentional cessation for at least 24 hours in the past year [30]. Binge drinking was defined as having more than five drinks (males) or more than four drinks (females) on a single occasion within the last 30 days [31].

The survey was modified between 2002 and 2010. Questions about the location of cigarette purchases produced a large number of missing responses. The instrument was subsequently modified to include specific modes of tax-avoidant purchase (internet/mail, another person/street in NYC, in New York State (NYS) outside NYC, other state, Indian reservation, outside USA, duty free).

The household measure of income has also changed. In 2002, respondents were asked to provide their yearly household income. For 2010, respondents' poverty level was measured based on federal poverty level (FPL) thresholds (<200% FPL, 200–<400% FPL, ≥400% FPL), annual income thresholds used to estimate the number of people in poverty nationwide. To enable comparisons between 2002 and 2010, a new poverty variable for 2002 was created and estimated from the income variable. The estimation incorporated observations with partial information on income and corrects for observations with insufficient information to assign an income category.

The 2010 survey also included items to measure how many days per week and month cigarettes were smoked to more accurately measure CPD. To assess a key dimension of nicotine dependency, the 2010 survey asked “how soon after waking up do you smoke your first cigarette?”

Because the CHS uses a complex sampling design, analyses require the use of a stratifying variable and a weighting variable. The stratifying variable has 34 strata that represent neighborhoods derived from the United Hospital Fund (UHF) classification system [32]. An additional stratum was added in 2010 to represent the cell phone only sample. The weighting variable adjusts for probability of selection and poststratification. Poststratification is accomplished by weighting each record up to the population of the neighborhood (as defined by UHF), while taking into account the respondent's age, gender, and race. Starting in 2009, responses were also weighted to account for the distribution of the adult population comprising three telephone usage categories (landline only, landline and cell, cell only) using data from the 2008 New York City Housing and Vacancy Survey.

For each survey year, cases were required to have nonmissing values for at least three or more of the following variables in order to meet BRFSS guidelines for completeness: age, Hispanic status, race/ethnicity, marital status, education, employment, and phone (do you have more than one telephone in your household?). From the base sample of complete interviews in 2002 (*N* = 9, 674) and in 2010 (*N* = 8, 665), our final analytic sample included 2,113 smokers in 2002 and 1,141 smokers in 2010.

### 2.3. Statistical Analysis

Changes in the number and proportion of Nondaily, light, and heavy smokers were assessed by comparing 2002 and 2010 data. Additionally, to compare characteristics and behaviors that were associated with being a Nondaily smoker, proportions were calculated for each variable of interest using the 2002 and 2010 datasets. Variables included were selected based on *a priori* knowledge of characteristics and behaviors associated with Nondaily smoking [1, 7, 14, 24]: age, race/ethnicity, gender, borough of residence, education level, and income; we also examined quit attempts, healthcare professional advice regarding cessation, having a smoke-free home policy, time to first cigarette after waking, source of last cigarette purchased (carton, pack, or loose single), and location of last cigarette purchase. Source and location of last cigarette were used to assess smokers' purchasing patterns for evidence of tax-avoidant purchases. Chi-square tests were used to identify significant variation between 2002 and 2010 among the stratifying variables. Significant chi-squares were followed up with pairwise comparisons between 2002 and 2010 prevalence estimates using *t* tests. A multivariable analysis was used to test the significance of changes in characteristics of the Nondaily smoker population between 2002 and 2010. Variables found to be significant in bivariate analysis (*P* < 0.05) were included in the multivariable model.

Next, using 2010 data, we compared demographic and smoking-related characteristics of Nondaily smokers to those of light smokers and heavy smokers, separately, in order to identify significant differences in these populations. Chi-square tests were used to identify significant variation between groups; significant chi-square tests were followed up with pairwise comparisons using *t*-tests. All differences were considered significant at *P* < 0.05.

A multivariable logistic regression model was used to identify characteristics associated with Nondaily smoking in 2010. The dependent variable was a dichotomous indicator of Nondaily (coded as 1) versus daily smoking (coded as 0). Independent variables found to be significant in bivariate analysis (*P* < 0.05) were considered candidates in the multivariable model. Potential confounding variables were also included in the model based on *a priori *knowledge of characteristics and behaviors associated with Nondaily smoking [1, 7, 12, 14].

To assess effect modification, we also included interaction terms derived from previous research. Several studies suggest that the relationship between Nondaily smoking and education may be modified by sex, and the association between Nondaily smoking and binge drinking may be modified by race [11, 17, 23]. Thus, we included terms for these interactions in the model. An interaction term between tax-avoidant purchasing behavior and smoking rules in the home was also included to help explain the relationship between Nondaily smoking and home smoking rules, which has been found in previous research [7]. The final model included three interaction terms: sex x education level; racial/ethnicity X binge drinking; and having a smoke-free home policy X tax-avoidant purchasing behavior. Significant effects were retained in the final model. Model fit was assessed using the likelihood ratio test. Adjusted odds ratios (AORs) and corresponding 95% confidence intervals (CIs) and *P* values were derived from the final models.

All analyses were conducted using the survey procedures in SAS v.9.1 (SAS Institute Inc., Cary, NC) and SAS-callable SUDAAN v.10 (Research Triangle Institute, Research Triangle Park, NC) to account for the complex survey design, incorporate the survey weights and age standardize estimates. In the descriptive analyses, all estimates were standardized to the US 2000 standard population using four age strata (18–24; 25–44; 45–64; 65+). All analyses (descriptive and multivariable) were weighted to the NYC adult population.

## 3. Results

### 3.1. Changes in Nondaily Smoking over Time

**Figure 1:**
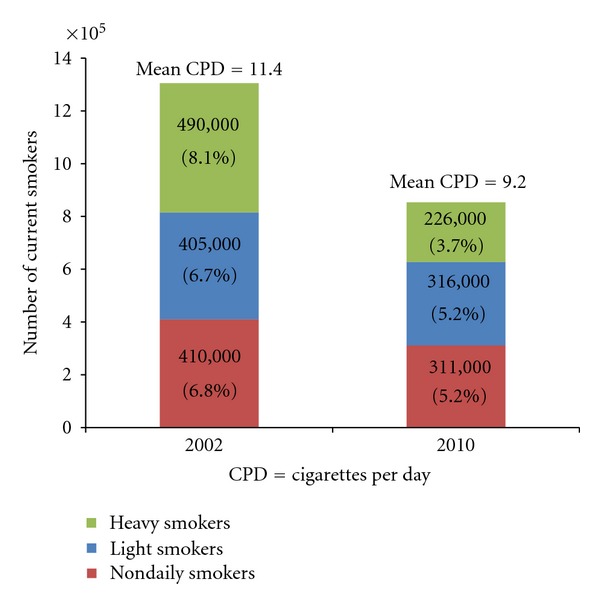
*Types of smokers, 2002 versus 2010. *Source: Community Health Survey 2002, 2010. Data are age-standardized to the US 2000 Standard Population. Estimated number and proportions are among the total NYC population aged 18 years and older.

Between 2002 and 2010, NYC saw a 35% overall decline in adult smoking prevalence in NYC, from 22% to 14% (data not shown). Since 2002, the number of heavy smokers in NYC has declined by more than half, from an estimated 490,000 in 2002 (representing 8% of the adult population) to about 226,000 in 2010 (representing 4% of the adult population) (Figure 1). The number of Nondaily smokers declined by about one-quarter from an estimated 410,000 in 2002 (representing 7% of the adult population) to about 311,000 in 2010 (representing 5% of the adult population). The decrease in the number of light smokers was similar to that of Nondaily smokers. In 2002 and 2010, the majority of current smokers was either Nondaily or light smokers (62% and 73%, resp.).

In 2002, about one-third (31%) of adult smokers in NYC reported smoking only on some days (Table 1); that percentage significantly increased to 36% in 2010 (*P* = 0.050). Across both years (2002 and 2010), Nondaily smokers were most likely to be between 25 and 44 years old (range of 52-53% across years), white (41% in both years), and have at least some college education (55–61%). The majority of participants reported making a quit attempt in the last 12 months (range of 73-74% across years) and 27–29% engaged in binge drinking. Nondaily smokers were more likely to make tax-avoidant purchases in 2010 (29%) than in 2002 (12%).

Two demographic characteristics of Nondaily smokers differed across years (Table 1). While the proportion of Nondaily smokers living in the borough of Manhattan declined from 29% in 2002 to 16% in 2010 (*P* < 0.001), the proportion that resides in Queens increased (23% in 2002 versus 32% in 2010, *P* = 0.022). Finally, the proportion of Nondaily smokers in the lowest income category increased from 2002 to 2010 (33% versus 46%, *P* = 0.004), while the proportion in the middle income category declined (34% versus 22%, *P* = 0.004). The proportion of Nondaily smokers who did not allow smoking in the home increased (41% versus 52%, *P* = 0.001).

Multivariable logistic regression analyses designed to test if the changes in these characteristics were significant between 2002 and 2010 showed that only the decrease in residents in Manhattan remained significant after controlling for demographic characteristics and smoking behaviors (AOR = 0.3, 95% CI (0.10, 0.89); data not shown). Thus, there were fewer Nondaily smokers in Manhattan in 2010 than in 2002.

### 3.2. Characteristics Associated with Nondaily Smoking in 2010

Several significant differences in demographic and smoking characteristics were found between Nondaily, and light or heavy smokers in 2010 (Table 2). Whites were less likely to be Nondaily smokers than heavy smokers (41% versus 54%, *P* < 0.05), while blacks were more likely to be Nondaily smokers than heavy smokers (25% versus 10%, *P* < 0.05). Males were also more likely to be Nondaily smokers than heavy smokers (51% versus 66%, *P* = 0.029). The highest rate of quit attempts was among Nondaily smokers, while the percent of smokers advised to quit by a healthcare professional was lowest among Nondaily smokers in comparison to light and heavy smokers (44% versus 60% and 65%; *P*s < 0.01). Compared to heavy smokers, more Nondaily smokers reported having rules about smoking in the home (52% versus 27%, *P* < 0.001). Most Nondaily smokers (79%) reported smoking their first cigarette of the day more than one hour after waking, in comparison to light smokers (41%) and heavy smokers (15%), *P* < 0.001. Significantly fewer Nondaily smokers smoked their last cigarette from a carton, as compared to heavy smokers (8% versus 35%, *P* < 0.001), and from a pack, as compared to light smokers (66% versus 77%, *P* < 0.001). 

### 3.3. Correlates of Nondaily Smoking

The results of the multivariable model are reported in Table 3. In the adjusted model, Nondaily smokers were more likely to smoke their first cigarette of the day more than one hour after waking (AOR = 8.8, 95% CI (5.38–14.27)). Other variables associated with being a Nondaily smoker included buying single loose cigarettes (AOR = 3.5, 95% CI (1.72–7.08)), and making a quit attempt in the last year (AOR = 2.3, 95% CI (1.36–3.96)). Our results further suggest that the relationship between Nondaily smoking and having rules limiting smoking in the home varies as a function of cigarette purchasing behavior (AOR = 6.57, 95% CI (1.96–22.01)). Among smokers who try to avoid NYC cigarette taxes, Nondaily smoking was more common among those with rules limiting smoking in the home (AOR = 3.51, 95% CI (2.76–4.47)) but not for non-tax-avoidant smokers (AOR = 0.54, 95% CI (0.20–1.41)). Similarly, race moderated the relationship between binge drinking and Nondaily smoking (OR = 4.62, 95% CI (1.59–13.48)). The odds of being a Nondaily smoker was higher for racial/ethnic minorities who engage in binge drinking (AOR = 2.06, 95% CI (1.61–2.64)) but this did not hold for whites (AOR = 0.45, 95% CI (0.20–1.01)). There was also evidence that sex moderated the relationship between Nondaily smoking and educational attainment (OR = 2.49, 95% CI (0.91–6.82), *P* = .08). Males with at least some college education had more than twice the odds of being a Nondaily smoker (AOR = 2.6, 95% CI (1.23–5.43)), while the odds of being a Nondaily smoker among females did not vary by education (AOR = 1.03, 95% CI (0.80–1.35)). Income was collinear with education and excluded from the model. 

## 4. Discussion

### 4.1. Key Results and Main Conclusions

Nondaily smokers now account for more than one-third of all adult smokers in NYC, and this proportion is much higher than the proportion seen nationally [6]. We noted a significantly higher proportion of Nondaily smokers were Queens residents in 2010 than in 2002, and rates of Nondaily smoking rose faster among New Yorkers in the lowest income category during that period. Together these trends suggest an increase in Nondaily smoking among lower-income New Yorkers—a departure from earlier studies that have associated Nondaily smoking with higher income and education levels [19, 24, 33]. Alongside these demographic shifts, there was an increase in purchasing behaviors associated with tax avoidance between 2002 and 2010. Price increases on cigarettes resulting from higher taxes seem the most plausible explanation for this shift. Within the context of NYC's tobacco control efforts, this trend suggests that low-income and price-sensitive smokers may be consuming fewer cigarettes in response to higher prices. 

Binge drinking has been explored in previous studies, particularly as it relates to college students and social smoking [9, 11, 12, 23]. The small sample size of 18–24-year olds in our study prevents us from detecting and exploring trends among this age group. However, we documented that nearly one-third of Nondaily smokers have engaged in binge drinking and found that Nondaily smokers were more likely to be racial/ethnic minorities in comparison to heavy smokers. 

Our results suggest that Nondaily smokers may be a “ready to quit” population that is less nicotine dependent than other groups of smokers [1, 7, 26]. Compared to light and heavy smokers, Nondaily smokers were more likely to have tried to quit smoking and to wait longer to smoke their first cigarette, suggesting a lower level of nicotine dependency [34]. Nondaily smokers were also more likely to purchase single loose cigarettes, and those who banned smoking in the home appear to comprise a price-sensitive subsgroup. Healthcare providers appear to be overlooking this group; however, Nondaily smokers were less likely to be advised to quit smoking by a healthcare professional. 

Many researchers have noted that Nondaily smoking may increase as a result of expanding tobacco control legislation and cigarette price increases [2, 4, 35, 36]. The Chaiton and Cohen hypothesis regarding the “softening” of the smoking population may be a useful framework for interpreting our findings in this regard [37]. Although more research is needed to measure nicotine addiction among Nondaily smokers in NYC, our results are consistent with the “softening” hypothesis. We saw a shift among the smoking population away from heavy daily smoking toward Nondaily smoking; we noted that low-income New Yorkers comprised a larger proportion of the Nondaily smoking population in 2010 as compared to 2002; we saw that Nondaily smokers were more likely than daily smokers to purchase single cigarettes than a pack. These factors point to the possibility that NYC's smoking population may be reducing their cigarette consumption in response to NYC's comprehensive tobacco control plan and transitioning toward becoming persistent Nondaily smokers.

### 4.2. Limitations

The NYC CHS is a population-based survey of smokers that relies upon self-reported data. Its cross-sectional design limits our ability to draw causal inferences. However, the surveys were large, conducted in multiple languages, and weighted to ensure they are representative of the NYC population; respondent opinions correlate well with both observed declines in smoking and predictions from the literature [5, 6]. In our analyses, a small number of smokers who reported smoking on some days only were classified as heavy smokers due to their high levels of consumption (23 cases total between 2002 and 2010). However, results from an exploratory analysis in which the 23 cases were classified as Nondaily smokers did not differ from the results presented here; thus, this classification did not impact our results. Finally, the change in the CPD imputation method between 2002 and 2010 could have contributed to decreases in mean CPD between 2002 and 2010.

### 4.3. Future Directions

New York has a greater percentage of Nondaily smokers than the US as a whole—a trend also seen in California, another jurisdiction with a strong tobacco control program [38]. This shift in smoking trends indicates that as tobacco control efforts spread around the nation, the phenomenon of Nondaily smoking may increase. Accordingly, new cessation policies and educational messaging may need to be tailored to this growing subpopulation of Nondaily smokers. NYC now has the highest cigarette excise taxes in the nation and comprehensive smoke-free air laws that prohibit smoking in bars, restaurants, and other public spaces. These distinctive environmental aspects may render our findings unique to NYC. Further research is needed to assess whether other jurisdictions with less comprehensive tobacco control policies are experiencing similar trends. 

 In bivariate analysis, we found that among Nondaily smokers a higher proportion were Queens residents in 2010 compared to 2002. Queens is home to many recent immigrants; 48% of the population is foreign-born, compared to 22% of the NYC population as a whole [39]. Because previous research has documented that Nondaily smoking is common among this group, particularly among Hispanic immigrants [21, 40], increasing rates of Nondaily smoking in Queens could reflect recent immigration in that borough. It should be noted that the changes in Queens were not significant in the full multivariable model, suggesting the decline was confounded by another predictor. However, our data do not allow us to identify immigration trends. Further research will be needed to explore this hypothesis in more detail. 

Our findings also identified low levels of cessation advice by healthcare providers in this population, indicating that new questions may be necessary to screen effectively for Nondaily smoking in this setting. The question “are you a smoker?” may not resonate with Nondaily smokers; it may be more effective to ask if a patient smokes cigarettes every day, some days, or not at all. New Joint Commission guidelines, scheduled to take effect in 2012, encourage a similar approach, stipulating that healthcare providers screen patients for tobacco use in the past 30 days to assess and document their patients' smoking status [41]. Providers should adopt these new guidelines to better identify and treat Nondaily smokers. 

Because Nondaily smokers may perceive themselves at lower risk for adverse health effects [40], and in view of findings here and in other studies that healthcare providers may not be routinely advising Nondaily smokers to quit [7], more research is needed on how to effectively assess and convey the health risks of Nondaily smoking. Furthermore, because common cessation aids may not be indicated for Nondaily smokers, incorporating assessments of nicotine dependency would be instrumental to future studies. 

Many of the studies that have sought to categorize different subgroups of Nondaily smokers have often relied on qualitative studies that have been limited to specific populations, such as college students [16, 24–26]. Clear definitions of subgroups that account for both the smoking characteristics and behaviors documented in these smaller studies as well as broader trends seen in population-based studies [7, 40] would allow for better tailoring of antitobacco efforts toward the needs of Nondaily smokers. Price-sensitive smokers may be one such group; however, identifying the psychosocial characteristics of price-sensitive smokers could allow for better targeting of antitobacco interventions to their needs. Additional studies focusing on social smoking, particularly among young people, and on the prevalence of Nondaily smoking among recent immigrants to NYC would assist in the development of more effective interventions.

## Figures and Tables

**Table 1:** Characteristics of Nondaily adult smokers aged 18 years and older, by select demographics—New York City Community Health Survey 2002 versus 2010.

	2002		2010		Chi-square test
	%	95% CI (LCI, UCI)	%	95% CI (LCI, UCI)	*P* value
Nondaily smokers overall	30.6	(28.1, 33.3)	35.6	(31.5, 40.0)	0.049
Age group					
18–24	17.4	(13.8, 21.7)	14.6	(9.5, 22.0)	
25–44	53.3	(48.4, 58.2)	52.0	(44.8, 59.1)	
45–64	23.6	(19.8, 27.9)	26.8	(21.4, 32.9)	0.723
65+	5.7	(4.0, 8.0)	6.6	(4.5, 9.6)	
Race/Ethnicity					
White non-Hispanic	40.7	(35.8, 45.9)	41.3	(34.6, 48.4)	
Black non-Hispanic	24.5	(20.4, 29.2)	25.0	(20.0, 30.8)	
Hispanic	24.3	(20.5, 28.6)	27.2	(21.5, 33.6)	0.516
Other non-Hispanic	10.4	(7.4, 14.4)	6.6	(3.8, 11.1)	
Male	49.0	(44.0, 54.1)	50.6	(43.8, 57.3)	0.955
Borough of residence					
Bronx	16.4	(12.9, 20.6)	16.7	(12.3, 22.2)	
Brooklyn	26.6	(22.5, 31.2)	30.2	(24.7, 36.5)	
Manhattan	28.7	(24.3, 33.5)	15.6*	(11.3, 21.1)	0.005
Queens	23.0	(18.6, 28.0)	32.4*	(26.2, 39.2)	
Staten Island	5.4	(3.8, 7.5)	5.1^*∧*^	(2.7, 9.4)	
HS Graduate or Less (among adults aged 25+)	45.1	(40.0, 50.3)	39.2	(32.8, 46.0)	0.348
Income From All Sources (% federal poverty level)					
<200 FPL	33.4	(28.2, 39.0)	46.4*	(39.4, 53.5)	
200–<400 FPL	33.7	(28.4, 39.3)	21.6*	(16.2, 28.4)	0.016
≥400 FPL	33.0	(28.1, 38.3)	32.0	(25.5, 39.2)	
Smoking Cessation (past 12 months)					
Tried to quit smoking	73.9	(69.1, 78.3)	73.4	(67.1, 79.0)	0.776
Received provider advice to quit smoking	43.9	(38.9, 48.9)	43.5	(37.1, 50.1)	0.566
Smoking is not allowed in the home	40.9	(35.8, 46.2)	52.3	(46.0, 58.6)	0.001
Last cigarette purchased from tax-avoidant location	12.1	(9.1, 16.0)	29.3	(22.7, 36.8)	<.001
Binge drinking (last 30 days)	27.2	(23.1, 31.7)	28.7	(23.0, 35.1)	0.774

*Significant difference between 2002 and 2010, *P* < .05; indicated on variables with more than two levels.

^*∧*^Estimate's Relative Standard Error (a measure of estimate precision) is greater than 30% or the sample size is too small, making the estimate potentially unreliable.

n/c: not calculated because one or more estimates is unreliable.

We present only one category for dichotomous variables to eliminate redundancy in the table.

**Table 2:** Characteristics of Nondaily, light and heavy smokers, current adult smokers aged 18 years and over—New York City Community Health Survey, 2010.

	Nondaily smoker	Light smoker	Heavy smoker	*P* value
	%	%	%	
Overall	35.6	37.0	27.4*	0.009
Mean cigarettes per day (SE)	1.8 (0.1)	7.1 (0.2)*	23.4 (1.0)*	<.001
Age group				
18–24	14.6	16.4	5.0^*∧*^	0.061
25–44	52.0	45.4	47.5	
45–64	26.8	30.5	36.3	
65+	6.6	7.7	11.2	
Race/Ethnicity				
White non-Hispanic	41.3	31.3*	54.0*	<.001
Black non-Hispanic	25.0	23.0	10.4*	
Hispanic	27.2	36.8	24.0	
Other non-Hispanic	6.6	9.0	11.6	
Male	50.6	47.1	66.4*	0.029
Borough of residence				
Bronx	16.7	18.5	15.5	0.428
Brooklyn	30.2	27.6	29.6	
Manhattan	15.6	22.7	19.3	
Queens	32.4	27.3	28.3	
Staten Island	5.1^*∧*^	3.9	7.2	
HS graduate or less (among adults aged 25+)	39.2	44.3	48.5	0.020
Income (% federal poverty level)				
<200 FPL	46.4	53.6	49.4	0.207
200–<400 FPL	21.6	16.8	13.5*	
≥400 FPL	32.0	29.7	37.1	
Smoking Cessation (past 12 months)				
Tried to quit smoking	73.4	51.4*	54.8*	0.001
Received provider advice to quit	43.5	59.6*	64.8*	<.001
Smoking is not allowed in the home	52.3	44.2	27.1*	<.001
Time to first cigarette				
Within 60 minutes	21.5	59.0*	85.5*	<.001
More than 1 hour	78.5	41.0	14.5	
Source of last cigarette				
Carton	7.8	10.4	35.4*	<.001
Pack	66.1	76.6*	60.2	
Single/loosie/bummed/roll own	26.1	13.0*	4.4^*∧*^	
Last cigarette purchased from tax-avoidant location	70.7	85.3*	60.8	<.001
Binge Drinking (last 30 days)	28.7	24.9	38.0	0.503

*Significantly different from Nondaily smokers, *P* < .05. See Section 2 for descriptions and definitions of smoker types.

^*∧*^Estimate's Relative Standard Error (a measure of estimate precision) is greater than 30% or the sample size is too small, making the estimate potentially unreliable.

We present only one category for dichotomous variables to eliminate redundancy in the table.

**Table 3:** Multivariable logistic analyses predicting Nondaily smoking versus daily smoking among current smokers, aged 18 years and over—New York City, 2010.

	Main effects model		Interaction effects model	
	Adjusted odds ratio	(95% CI) LCI, UCI	Adjusted Odds Ratio	(95% CI) LCI, UCI
Age Group				
18–44	0.83	(0.49, 1.40)	0.87	(0.51, 1.50)
45+	Ref.		Ref.	
Borough of Residence				
Brooklyn	1.10	(0.55, 2.22)	1.06	(0.52, 2.19)
Manhattan	0.66	(0.30, 1.48)	0.63	(0.27, 1.47)
Queens	1.03	(0.48, 2.22)	1.00	(0.45,2.20)
Staten Island	1.17	(0.42, 3.23)	1.02	(0.32, 3.20)
Bronx	Ref.		Ref.	
Time to first cigarette of the day				
More than 1 hour after waking up	8.32*	(2.05, 13.70)	8.76*	(5.38, 14.27)
Within 60 minutes of waking up	Ref.		Ref.	
Last cigarette purchased				
Carton	0.39*	(0.17, 0.91)	0.41*	(0.19, 0.90)
Single/loosie/bummed/rolled-your-own	3.61*	(1.79, 7.28)	3.49*	(1.72, 7.08)
Pack	Ref.		Ref.	
Cessation attempts in the past year				
Tried to quit smoking	2.15*	(1.28, 3.60)	2.32*	(1.36, 3.96)
Did not try to quit smoking	Ref.		Ref.	
Sex				
Male	0.87	(0.53, 1.40)	0.51	(0.22, 1.16)
Female	Ref.		Ref.	
Race/ethnicity				
All other races	1.11	(0.64, 1.91)	0.66	(0.3, 1.24)
White	Ref.		Ref.	
Rules about smoking in home				
Smoking is not allowed	1.10	(0.68, 1.79)	0.74	(0.43, 1.28)
Smoking is allowed in some or all areas	Ref.		Ref.	
Education (among adults aged 18+)				
Some college or more	1.75*	(1.06, 2.91)	1.03	(0.80, 1.35)
High school grad or less	Ref.		Ref.	
Last cigarette purchased				
Outside NYC/tax-avoidant	1.51	(0.80, 2.84)	0.54	(0.20, 1.41)
In New York City/nontax-avoidant	Ref.		Ref.	
Binge drinker				
Yes	1.06	(0.60, 1.88)	0.45*	(0.20, 1.01)
No	Ref.		Ref.	

Smoking not allowed in the home X tax-avoidant	—	—	6.57*	(1.96, 22.01)
All other race X binge drinker	—	—	4.62*	(1.59, 13.48)
Sex X some college or more education	—	—	2.49^†^	(0.91, 6.82)

^†^ *P* < .10, *P < .05.

## References

[1] Schane RE, Glantz SA, Ling PM (2009). Nondaily and social smoking: an increasingly prevalent pattern. *Archives of Internal Medicine*.

[2] Shiffman S (2009). Light and intermittent smokers: background and perspective. *Nicotine & Tobacco Research*.

[3] Burns DM, Major JM, Anderson CM, Vaughn JW (2003). Changes in cross-sectional measures, numbers of cigarettes smoked per day, and time to first cigarette—California and national data. *Smoking and Tobacco Control Monograph 15: Those Who Continue to Smoke*.

[4] Luoto R, Uutela A, Puska P (2000). Occasional smoking increases total and cardiovascular mortality among men. *Nicotine & Tobacco Research*.

[5] Centers for Disease Control and Prevention (2004). Cigarette smoking among adults—United States, 2002. *Morbidity and Mortality Weekly Report*.

[6] Centers for Disease Control and Prevention (2011). Vital signs: current cigarette smoking among adults aged ≥18 years—United States, 2005–2010. *Morbidity and Mortality Weekly Report*.

[7] Tong EK, Ong MK, Vittinghoff E, Pérez-Stable EJ (2006). Nondaily smokers should be asked and advised to quit. *American Journal of Preventive Medicine*.

[8] Tindle HA, Shiffman S (2011). Smoking cessation behavior among intermittent smokers versus daily smokers. *American Journal of Public Health*.

[9] Rutten LJ, Augustson EM, Doran KA, Moser RP, Hesse BW (2009). Health information seeking and media exposure among smokers: a comparison of light and intermittent tobacco users with heavy users. *Nicotine & Tobacco Research*.

[10] Levy DE, Biener L, Rigotti N (2009). The natural history of light smokers: a population-based cohort study. *Nicotine & Tobacco Research*.

[11] Berg CJ, Lust KA, Sanem JR (2009). Smoker self-identification versus recent smoking among college students. *American Journal of Preventive Medicine*.

[12] Levinson AH, Campo S, Gascoigne J, Jolly O, Zakharyan A, Tran ZV (2007). Smoking, but not smokers: identity among college students who smoke cigarettes. *Nicotine & Tobacco Research*.

[13] Schane RE, Ling PM, Glantz SA (2010). Health effects of light and intermittent smoking: a review. *Circulation*.

[14] Hassmiller KM, Warner KE, Mendez D, Levy DT, Romano E (2003). Nondaily smokers: who are they?. *American Journal of Public Health*.

[15] Hennrikus D, Jeffery R, Lando H (1996). Occasional smoking in a Minnesota working population. *American Journal of Public Health*.

[16] Zhu SH, Sun J, Hawkins S, Cummins S (2003). A population study of low-rate smokers: quitting history and instability over time. *Health Psychology*.

[17] Trinidad DR, Pérez-stable EJ, Emery SL, White MM, Grana RA, Messer KS (2009). Intermittent and light daily smoking across racial/ethnic groups in the United States. *Nicotine & Tobacco Research*.

[18] 2008 PHS Guideline Update Panel, Liaisons, and Staff (2008). Treating tobacco use and dependence: 2008 update—U.S. public health service clinical practice guideline
executive summary. *Respiratory Care*.

[19] Wortley PM, Husten CG, Trosclair A, Chrismon J, Pederson LL (2003). Nondaily smokers: a descriptive analysis. *Nicotine & Tobacco Research*.

[20] Fagan P, Rigotti N (2009). Light and intermittent smoking: the road less traveled. *Nicotine & Tobacco Research*.

[21] Reitzel LR, Costello TJ, Mazas CA (2009). Low-level smoking among Spanish-speaking Latino smokers: relationships with demographics, tobacco dependence, withdrawal, and cessation. *Nicotine & Tobacco Research*.

[22] White HR, Bray BC, Fleming CB, Catalano RF (2009). Transitions into and out of light and intermittent smoking during emerging adulthood. *Nicotine & Tobacco Research*.

[23] Harrison EL, Desai RA, McKee SA (2008). Nondaily smoking and alcohol use, hazardous drinking, and alcohol diagnoses among young adults: findings from the NESARC. *Alcoholism*.

[24] Evans NJ, Gilpin E, Pierce JP (1992). Occasional smoking among adults: evidence from the California Tobacco Survey. *Tobacco Control*.

[25] Shiffman S, Kirchner TR, Ferguson SG, Scharf DM (2009). Patterns of intermittent smoking: an analysis using ecological momentary assessment. *Addictive Behaviors*.

[26] Nguyen QB, Zhu S (2009). Intermittent smokers who used to smoke daily: a preliminary study on smoking situations. *Nicotine & Tobacco Research*.

[27] New York City Department of Health and Mental Hygiene Health department announces an eight-year decline in smoking-related deaths in New York City as smoking remains at an all-time low. http://www.nyc.gov/html/doh/html/pr2010/pr050-10.shtml.

[28] New York City Department of Health and Mental Hygiene Community health survey: methodology. http://www.nyc.gov/html/doh/html/survey/chs-methods.shtml.

[29] Centers for Disease Control and Prevention (CDC) *Behavioral Risk Factor Surveillance System Survey Questionnaire*.

[30] Gilpin EA, Pierce JP, Farkas AJ (1997). Duration of smoking abstinence and success in quitting. *Journal of the National Cancer Institute*.

[31] New York City Department of Health and Mental Hygiene (2007). Who’s still smoking? Cigarette use among adults in New York City. *Vital Signs*.

[32] New York City Department of Health and Mental Hygiene New York City United Hospital Fund (UHF) Neighborhoods and Zip Codes. http://www.nyc.gov/html/doh/downloads/pdf/survey/uhf_map_100604.pdf.

[33] Husten CG (2009). How should we define light or intermittent smoking? Does it matter?. *Nicotine & Tobacco Research*.

[34] Mooney ME, Johnson EO, Breslau N, Bierut  LJ, Hatsukami DK (2011). Cigarette smoking reduction and changes in nicotine dependence. *Nicotine & Tobacco Research*.

[35] Korhonen T, Broms U, Levälahti E, Koskenvuo M, Kaprio J (2009). Characteristics and health consequences of intermittent smoking: long-term follow-up among Finnish adult twins. *Nicotine & Tobacco Research*.

[36] Pierce JP, White MM, Messer K (2009). Changing age-specific patterns of cigarette consumption in the United States, 1992–2002: association with smoke-free homes and state-level tobacco control activity. *Nicotine & Tobacco Research*.

[37] Chaiton MO, Cohen JE, Frank J (2008). Population health and the hardcore smoker: Geoffrey Rose revisited. *Journal of Public Health Policy*.

[38] California Department of Public Health, California Tobacco Program (2009). *California Tobacco Control Update 2009: 20 Years of Tobacco Control in California*.

[39] US Census Bureau State and County Quickfacts. http://quickfacts.census.gov/qfd/states/36/36081.html.

[40] Ackerson LK, Viswanath K (2009). Communication inequalities, social determinants, and intermittent smoking in the 2003 Health Information National Trends Survey. *Preventing Chronic Disease*.

[41] Joint Commission http://www.jointcommission.org/specifications_manual_for_national_hospital_inpatient_quality_measures/.

